# Stratification of Hepatocellular Carcinoma Risk Following HCV Eradication or HBV Control

**DOI:** 10.3390/jcm10020353

**Published:** 2021-01-19

**Authors:** Pierre Nahon, Erwan Vo Quang, Nathalie Ganne-Carrié

**Affiliations:** 1 AP-HP, Hôpital Avicenne, Liver Unit, 93000 Bobigny, France; erwan.voquang@gmail.com (E.V.Q.); nathalie.ganne@aphp.fr (N.G.-C.); 2 “Equipe labellisée Ligue Contre le Cancer”, Université Paris 13, Sorbonne Paris Cité, 93206 Saint-Denis, France; 3 INSERM UMR-1138, Functional Genomics of Solid Tumours, Centre de Recherche Les Cordeliers, 75006 Paris, France

**Keywords:** viral hepatitis, hepatocellular carcinoma, antivirals, risk scores, surveillance

## Abstract

Hepatocellular carcinoma (HCC) incidence has dramatically decreased in patients infected with HCV and HBV due to the widespread use of highly effective antiviral agents. Nevertheless, a substantial proportion of patients with advanced fibrosis or cirrhosis following HCV clearance of in case of HBV control whatever the stage of fibrosis remains at risk of liver cancer development. Cancer predictors in these virus-free patients include routine parameters estimating coexisting comorbidities, persisting liver inflammation or function impairment, and results of non-invasive tests which can be easily combined into HCC risk scoring systems. The latter enables stratification according to various liver cancer incidences and allocation of patients into low, intermediate or high HCC risk probability groups. All international guidelines endorse lifelong surveillance of these patients using semi-annual ultrasound, with known sensibility issues. Refining HCC prediction in this growing population ultimately will trigger personalized management using more effective surveillance tools such as contrast-enhanced imaging techniques or circulating biomarkers while taking into account cost-effectiveness parameters.

## 1. Introduction

The widespread implementation of anti-HBV and anti-HCV therapies has deeply modified the course of chronic viral liver diseases, of which hepatocellular carcinoma (HCC) has become the leading cause of death [1]. The main goal of anti-HBV therapy using nucleos(t)ide analogues (NUCs) in patients with chronic active hepatitis B is limiting the progression of liver disease through long-term suppression of HBV viral load, a circumstance during which numerous studies have reported a reduction in HCC incidence [2]. In the vast majority of patients with HCV infection, including those with extensive fibrosis or cirrhosis, direct-acting antivirals (DAAs) are associated with a sustained virological response (SVR) [3]; similarly, HCC incidence in individuals infected with HCV with extensive fibrosis or cirrhosis is decreased following viral eradication. Nevertheless, despite HCV clearance or HBV control, the risk of HCC is not abolished in all patients. It is thus recommended that patients infected with HCV with extensive fibrosis or cirrhosis following SVR and patients infected with HBV under NUCs (irrespective of fibrosis stage) participate in dedicated HCC surveillance programmes [4].

Over the past decades, numerous HCC risk scoring systems for stratifying HBV- or patients infected with HCV into various HCC risk classes have been proposed and validated [5]. However, most of these risk scores were designed prior to the widespread use of antiviral therapies and are now outdated since they assigned heavy weighting to virological parameters. More recently, new stratification models have been developed through dedicated multicentric efforts in the current era of HCV eradication or HBV control following antiviral treatment. This review will focus on these models and will highlight their potential role in personalized management of these patients, who now survive longer and bring new challenges for physicians.

## 2. Why Should We Stratify Patients with Viral-Induced Disease According to HCC Risk?

The goal of HCC surveillance programmes is the detection of liver tumours at the earliest stage possible, in order to allocate patients to curative procedures which has been shown to provide a survival benefit [6]. Although semi-annual liver ultrasound (US) is recommended as the first-line tool for HCC surveillance, its sensitivity for the detection of HCC tumours within the BCLC 0 or A stages is low, below 50% [7]. Given these concerns, there has been increased interest in the use of alternative imaging modalities that employ contrast-enhanced procedures, such as computed tomography (CT) and magnetic resonance imaging (MRI) [8]. For instance, it has been shown that MRI performed as routine surveillance in cirrhotic patients yielded a detection sensitivity of 84.8% for very early-stage (BCLC 0) HCC, significantly better than the 27.3% achieved using US [8]. In addition, new serum biomarkers that may improve sensitivity for early HCC detection have been a focus of interest in numerous studies but are still under exploration [9].

**Figure 1:**
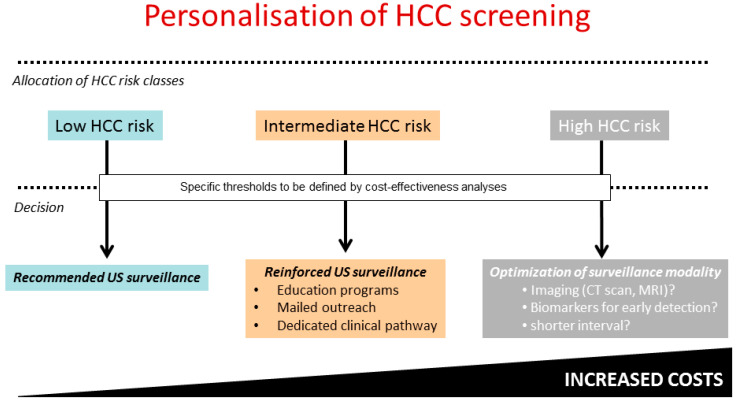
Potential application of hepatocellular carcinoma (HCC) risk stratification using scoring systems: reinforcement of surveillance programs in patients with the highest incidences to optimize screening uptake and efficacy. The optimal thresholds will depend upon the proportion of patients allocated to each class and on cost-effectiveness estimates.

However, implementing these new tools into surveillance programmes may not be cost-effective for all patients, particularly for those who have achieved HCV clearance or HBV control, in whom decreased annual HCC incidences are now well established [10]. In this context, highlighting patients with a particularly low HCC incidence while reinforcing screening programs in those who remain at higher risk are of paramount importance to trigger personalized management. The intensification of HCC surveillance programs in high-risk groups is indeed a timely challenge as it would not only improve compliance [11], which has been shown to increase access to HCC curative treatment and to improve overall survival in patients with viral cirrhosis [11], but would also overcome the pitfalls related to the low sensitivity of US examination [12]. For example, the use of an expensive but highly sensitive imaging technique such as MRI, which is able to detect small liver lesions, could be justified in populations with the highest risk of liver cancer [13] despite viral clearance or control, as reported by cost-effectiveness analyses performed in Asian patients infected with HBV with an annual HCC incidence above 3% [14]. Figure 1 shows a draft proposal for the potential application of HCC risk stratification in the setting of future personalized management in a way that might optimize the allocation of medical resources in a cost-effective fashion [13]. Achieving this goal will require the performance of dedicated studies in patients who have been stratified according to HCC risk; the definition of the optimal thresholds for allocation of patients into specific risk classes will depend upon the reported incidence of liver cancer following the successful implementation of antiviral therapy.

## 3. Decreased HCC Incidence in Patients with HCV-Related Extensive Fibrosis or Cirrhosis Following SVR

Following SVR, the risk of HCC is highest in patients with cirrhosis and is considered negligible in patients with mild or no fibrosis; HCC surveillance is not recommended for the latter group [4]. However, HCC may occur in patients with bridging fibrosis (METAVIR score F3) [15]. Whether based on studies conducted in the interferon treatment era or in the DAA treatment era, the absolute reduction in HCC risk is now well documented, but primary liver cancer still occurs over the long term at a rate that probably does not exceed 3% per year [16]. If all international guidelines endorse lifelong HCC surveillance following SVR in patients with cirrhosis [4,17,18], the case of patients with bridging fibrosis is debated with dedicated analyses suggesting a lack of cost-effectiveness in this population [10]. Nevertheless, European guidelines recommend surveillance of this subset [4,18], but not AASLD [17].

Ageing usually triggers the development of various comorbidities known to impact liver-related outcomes, including liver cancer [19]. Studies conducted in Japan during the interferon treatment era have reported HCC incidence as high as 15.9% after 15 years [20]. Similar observations were made in the West in patients with cirrhosis in whom longitudinal follow-up revealed a 1.39% yearly HCC incidence following SVR [21]. During the interferon treatment era, numerous studies convincingly showed that the risk of HCC decreased after SVR but remained sufficiently high to justify periodic screening [22]. Data obtained from European cohorts during prospective follow-up over a median of 8 years confirmed this benefit, with a 10-year cumulative HCC incidence reaching 5.1% [23]. When restricted to patients who were unambiguously diagnosed via biopsy with compensated cirrhosis (*n* = 1323, median follow-up 58 months), a prospective multicentre study reported a 5-year cumulative HCC incidence of 6.7% [24].

A similar evolution might be expected in patients who achieve SVR by means of DAA therapy. HCC incidence could even be higher in older patients with more pronounced liver disease and comorbidities compared to the available profiles reported for interferon-treated patients. The first available multicentre reports were restricted to the retrospective analysis of registries from the Veterans Affairs system, which reported a 71% lower risk of HCC in patients with HCV clearance [25,26,27]. This finding has since been prospectively confirmed through analysis of 9895 French patients with advanced fibrosis included in the ANRS CO22 Hepather cohort [28], where DAA treatment was associated with decreased HCC risk (adjusted HR = 0.66, 95% CI 0.46–0.93). Similarly, based on longitudinal cohorts recruited in tertiary hepatology units, a study performed in Europe reported the annual incidence of HCC as a function of liver function impairment in 2249 cirrhotic patients following DAA implementation. These analyses confirmed that there is a higher annual incidence in Child-Pugh Class B patients than in Class A patients (6.6% vs. 2.1%, respectively) [29]. In the ANRS CO12 CirVir cohort [30] which selected patients with biopsy-proven compensated cirrhosis, the performance of analyses that accounted for the differing characteristics of patients according to treatment allocation (interferon vs. DAAs) was possible. In this setting, confounders that may explain differences between patients from different therapeutic eras were taken into account and similar HCC incidences below 2% per year were reported based on rigorous analyses including inverse probability of treatment weighting (IPTW) method [31].

Overall, the magnitude of the decrease in HCC risk is similar regardless of the antiviral treatment regimen. However, due to the relatively short follow-up of patients who received DAAs, longer follow-up of patients and future updates of cohort studies should clarify the pattern of temporal evolution of HCC incidence. Until then, lifelong surveillance for HCC is recommended in patients with documented advanced fibrosis and cirrhosis, as it seems unlikely that the risk of liver cancer would eventually decrease over time to a point at which surveillance becomes unnecessary. Nevertheless, allocation of personalized screening procedures might be triggered by HCC risk stratification.

## 4. Identification of Patients Infected with HCV with Higher Residual HCC Risk Following SVR

Despite the low HCC incidence following SVR reported above, all patients with advanced fibrosis or cirrhosis do not have the same risk of developing HCC [32]. Until the availability of DAAs, various HCC scoring systems have been designed based on the combination of routine clinical features to stratify patients into various HCC risk classes, which in most cases did not consider SVR status [5]. Following the widespread implementation of these new regimens, a specific phenotype of patients who present a higher risk of liver cancer development despite SVR has been identified using simple routine parameters; it comprises various covariates, including higher rates of comorbidities (in particular linked to metabolic syndrome), persistent circulating necro-inflammatory markers and impaired liver function or persisting signs of portal hypertension [19,24,33]. Combining these variables using regression analysis enables allocation of patients into low-, moderate- or high-HCC risks. The most rigorous multicentric efforts using large training and validation sets are displayed in Table 1. Non-invasive assessment of liver disease, for instance, using liver stiffness measurement, can further add significant additional information, as recently shown in a cohort of patients with advanced chronic liver disease in whom liver stiffness measurement (LSM) and albumin levels following SVR could identify patients at higher or lower risk of HCC [34]. The sequential evolution of these non-invasive parameters may also be informative. Recently, the longitudinal assessment of serum fibrosis scores such as FIB-4 (Fibrosis-4 index) or APRI (aspartate aminotransferase to platelet ratio index) post-SVR was performed in more than 6000 patients in the VA system [35]. Cirrhotic patients who had persistently high FIB-4/APRI following SVR had the highest HCC incidence (between 3.3 and 6.5 per 100 PY), while the risk of HCC decreased in those who experienced a decline in FIB-4/APRI over time (0.6 to 2.8 per 100 PY). Similarly, changes in LSM following SVR in patients with advanced chronic liver disease could be incorporated into dedicated models that showed fair HCC predictive ability [36] These reports are still preliminary and require further exploration: there is currently no recommendation in clinical practice to adapt surveillance as a function of non-invasive fibrosis tests evolution following SVR.

**Figure 2:**
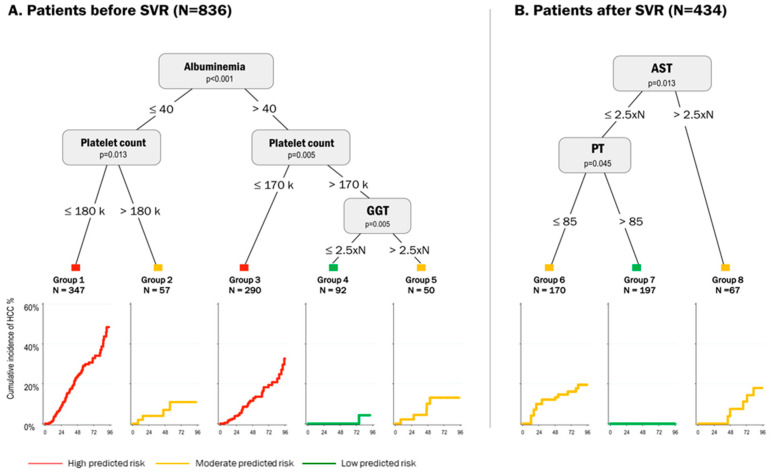
Example of the use of machine learning approach using decision tree analysis in patients infected with HCV with compensated cirrhosis as a function of SVR status in the ANRS CO12 CirVir cohort (adapted from Audureau et al. [44] with permission from the Authors). Five main predictors were identified by the algorithm, yielding eight groups (five before SVR [Panel **A**] and three following SVR [Panel **B**]) from various combinations of these predictors and demon-strating markedly contrasting risks of HCC, as shown by the corresponding curves at each end node.

Until recently, the stratification of HCC risk has been based only on simple scoring systems that combine routine clinical features without considering if these variable might operate in combination or independently [46,47]. In this context, more sophisticated approaches using machine learning approaches may in the future provide usable guidance for clinical practice [48]. This was recently applied to patients infected with HCV in whom risk was studied as a function of viral clearance. For instance, in the aforementioned ANRS CO12 CirVir cohort, which included cirrhotic patients during both the interferon era and the DAA era, machine learning approaches using decision tree analysis and random forest were applied to refine individualized predictions of HCC risk [44]. As expected, the clinical features associated with HCC differed in patients without SVR (past excessive alcohol intake, HCV genotype 1, platelet count, gamma glutamyltransferase (GGT), alpha-fetoprotein and albumin) and following SVR; prothrombin time and aspartate aminotransferase were predictors after SVR. The decision tree analysis revealed unsuspected interactions between variables and stratified patients in these two distinct clinical situations into 8 different phenotypes with different cancer risks (see Figure 2). In particular, SVR patients could be classified as having low or moderate HCC risk according to these simple biological parameters. Similarly, deep learning models were recently applied to data in the VA database [49]. In this context, use of a recurrent neural network (RNN) outperformed conventional models in identifying patients with the highest HCC risk regardless of their SVR status.

## 5. HCC Incidence in Patients with HBV-Related Liver Disease and Virosuppression Treated by NUCs

Entecavir (ETV), tenofovir disoproxil fumarate (TDF) and tenofovir alafenamide (TAF) are the three first-line NUCs with a high genetic barrier to resistance that are recommended by international treatment guidelines [2]. ETV and TDF maintain long-term viral suppression in over 95% of patients and can reverse the cirrhosis process [50]. Increased concentration of HBV DNA in serum is a well-established risk factor for the development of HCC among untreated chronic hepatitis B patients [51]. Additionally, elevated serum HBV DNA level is dose-dependently associated with an elevated risk of progression to cirrhosis, while cirrhosis itself is a strong risk factor for HCC [52]. This mechanism could explain the efficacy of NUCs in reducing the risk of HCC. The first studies to demonstrate the effectiveness of NUCs in reducing HCC risk in patients infected with HBV used lamivudine, a NUC with a low genetic barrier to resistance [53]. Since then, numerous studies have shown a reduced risk of HCC development in chronic HBV patients undergoing treatment with NUCs [54]. HCC incidence differs in patients with and without cirrhosis. Overall, in cirrhotic patients treated with ETV or TDF, HCC rates were approximately 4- to 5-fold higher than those in patients without cirrhosis, ranging from 0.9% to 5.4% in Asians and from 1.5% to 5.2% in Caucasians [55]. As in HCV infection, there is no recommendation to trigger personalized surveillance as a function of fibrosis non-invasive tests evolution following HBV control.

### 5.1. Patients with Cirrhosis

Once cirrhosis is diagnosed, NUCs are beneficial in preventing cirrhosis progression towards liver decompensation and reduce the risk of HCC development [56]. Most studies that investigated the efficacy of long-term NUC therapy in HCC reduction have focused on Asian populations.

In a multicentre, retrospective-prospective cohort study conducted in Taiwan, HCC incidence was 2.4% in the ETV group and 5.2% in the untreated group in the first 2.7 years; this corresponded to an HCC risk reduction of 60% (HR = 0.41; 95% CI 0.20–0.84) [57]. In previous Asian and European studies, the reported 5-year cumulative incidence of HCC in patients with compensated cirrhosis is 17%–39% in untreated patients, compared with 7%–18% in ETV groups [56,58,59]. Moreover, the HCC suppression effect in the ETV-treated group appears to be superior to that observed in LAM-treated cirrhotic patients [60]. However, in patients with cirrhosis, death or liver transplantation, which act as competing risks, were 50%–60% lower with ETV than with lamivudine after 3 years of follow-up; this may partially explain why HCC risk over the same time frame was similar between the two regimens [61]. Similarly, TDF was associated with a 77% reduction in the risk of HCC (HR, 0.23; 95% CI 0.56–0.92) in patients with cirrhosis; this group of patients experienced an 8-year cumulative incidence of HCC reaching 12.71% [62].

Papatheodoridis et al. studied the risk of HCC in a European, multicentre cohort study that included 1951 Caucasian chronic hepatitis B patients who received ETV or TDF [63]. Among them, 1205 (62%) patients who did not develop HCC within the first 5 years of therapy were followed for 5–10 (median 6.8) years. Long-term follow-up revealed that the yearly HCC incidence decreased after the first 5 years (3.22% in the first 5 years compared with 1.57% thereafter).

Patients with chronic hepatitis B virus (HBV) infection are at risk of HCC development even in the absence of cirrhosis [64]. Reducing the risk of progression to cirrhosis and liver-related complications, including HCC, is the main goal in managing these patients.

### 5.2. Patients without Cirrhosis

For noncirrhotic CHB patients, the mean annual incidence of HCC is lower and is reported to be 0.68% in patients undergoing treatment with NUCs and 2.97% in patients without treatment [65]. Conversely, in patients with cirrhosis, HCC rates are comparable in those receiving ETV and LAM regimens [60,61]. In noncirrhotic patients treated with ETV or TDF, annual HCC incidences ranged from 0.0% to 1.4% in Asian patients and from 0.1% to 1.0% in predominantly Caucasian populations [55]. The yearly HCC incidence during and after the first 5 years does not differ (0.49% versus 0.47%, respectively) [63].

### 5.3. Differences Between ETV and TDF

Two recent large Asian retrospective studies were the cause of some controversy. Using available adjusted data (multivariate or propensity-matched data), the risk of HCC among patients treated with ETV was 27% higher than that among patients treated with TDF [66]. Moreover, TDF treatment was associated with a significantly (20%) lower risk of HCC than was ETV treatment [67]. However, recent meta-analyses following numerous subsequent cohort studies found no significant difference between TDF and ETV in their association with HCC occurrence [68,69]. Ideally, randomized trials should be conducted to provide accurate answers.

## 6. HCC Risk Scoring Systems in Controlled Patients Infected with HBV

Similar to the case for patients infected with HCV with advanced fibrosis who reached SVR, numerous HCC risk scoring systems have been developed for CHB patients who are undergoing long-term NUC treatment. Initially, these scoring systems were constructed for untreated patients infected with HBV with chronic active HBV replication, while the more recently developed systems are specifically dedicated to patients under antiviral therapy. Most of these scoring systems were developed in Asians, and they often mix patients with and without cirrhosis [37,39,40,41,42,43]. Their components are described in Table 1. Another risk scoring system, the PAGE-B score [38], was developed in a European cohort. Usually applied to the Caucasian population, it allocates patients into three HCC classes according to simple, routinely measured parameters (platelets, age, gender). EASL endorses its application in noncirrhotic HBV patients to trigger HCC surveillance [4]. Patients in the low HCC risk group, schematically including men aged under 40 years and women aged under 70 years, with platelet counts above 200 G/L, had a negligible probability of HCC development. Recently, when the PAGE-B algorithm was applied to Asian populations, 25% of patients infected with HBV in Hong Kong were allocated to the low HCC risk group; their 5-year cumulative incidence of HCC was 0.6% (0.4%–0.8%) [70]. This classification achieved a negative predictive value of 99.5% in excluding patients without HCC development at 5 years. However, the persistent presence of detectable HBV DNA during NUC therapy is associated with HCC development [71], leading to the development of the new PAGE-B-DNA score. The latter combines features of the PAGE-B algorithm with the level of detected HBV DNA and has proven to be efficient in refining HCC risk stratification. 

## 7. Perspectives: Towards Universal HCC Risk Stratification and Precision Medicine?

As described above, the global annual HCC incidence in patients with controlled HBV infection or cured HCV in the case of advanced chronic liver disease ranges from 0.2 to 2.5%. These rates are similar to those observed in patients with cirrhosis due to nonviral causes, whether alcohol- or NASH-related [72,73], and can be predicted by identical nonviral features. It is thus tempting to develop universal scoring systems that could be applied regardless of the cause of the underlying liver disease. Recently, an international effort developed A global HCC risk scoring system in 17,374 patients; the population encompassed HBV-(75%) or patients infected with HCV without viral replication as well as patients with nonviral cause of liver disease who were recruited among 11 international prospective observational cohorts and randomized controlled trials [45]. The definite algorithm, called the aMAP score, selected older age, male sex, albumin-bilirubin and low platelet count as cancer predictors (see Table 1). This model was able to allocate patients to 3 distinct risk classes for 5-year HCC development irrespective of ethnicity and cause of liver disease, including a large low-risk group which accounted for ~45% of the overall population with an HCC probability of <0.2% per year.

The availability of different models has enabled so far to define several HCC risk classes that might define new surveillance strategies. The goal is not to identify patients who have “zero risk” following viral eradication or control; in any case, this would only be true at one time point in the patient’s lifetime since the probability of developing HCC typically increases over time: older age, the development of comorbidities and worsening of liver dysfunction are all cancer risk factors, justifying lifelong surveillance until further notice. In contrast, long-term follow-up of longitudinal cohorts has been able to highlight specific subgroups in which, despite their virus-free status, the persisting risk of liver cancer is sufficiently high to trigger personalized management through reinforced surveillance programs. The modelling approaches that have been used to achieve this goal are based on simple routine measurements that are readily available; these algorithms may possibly be enriched by the incorporation of circulating biomarkers [74] such as genetic variants [32,75,76] or epigenetic footprints [77,78], both of which have been shown to be specifically associated with higher HCC risk following virological control or clearance. Future areas for research in HCC risk stratification are thus extensive, and will ultimately optimize the allocation of medical resources in a cost-effective fashion.

## Figures and Tables

**Table 1:** Variables included in hepatocellular carcinoma (HCC) risk scores (or associated with HCC) developed in individuals with chronic hepatitis B and in patients infected with HCV with advanced fibrosis who achieved virological clearance or control status following antiviral therapy.

			Variables Included in HCC Risk Scores for Virologically Controlled Patients Infected with HBV									
Risk Score/Reference	Country or Area	Treatment	Host Factors			Liver Disease Activity		Cirrhosis/Fibrosis Parameters				
			Age	Gender	Others	AFP	AST or ALT	PT	PLT	LSM	Albumin	Bilirubin
**REACH-Bm [37]**	Korea	Entecavir	X	X						NA	X	
**PAGE-B [38]**	Europe	Entecavir/Tenofovir	X	X					X	NA		
**HCC-RESCUE [39]**	Korea	Entecavir	X	X						NA		
**APA-B [40]**	Taiwan	Entecavir	X			X			X	NA		
**CAMD [41]**	Taiwan/Hong Kong	Entecavir/Tenofovir	X	X	Diabetes					NA		
**mPAGE-B [42]**	Korea	Entecavir/Tenofovir	X	X					X	NA	X	
**AASL [43]**	Korea	Entecavir/Tenofovir	X	X						X	X	
			**Variables Included in HCC Risk Scores for Patients Infected with HCV with Advanced Chronic Liver Disease Who Achieved Sustained Virological Response (SVR)**									
			**Host factors**			**Liver disease activity**		**Cirrhosis/Fibrosis parameters**				
			**Age**	**Gender**	**Others**	**AFP**	**AST or ALT**	**PT**	**PLT**	**LSM**	**Albumin**	**Bilirubin**
**van der Meer 2017 [19]**	Europe	INF	X		Diabetes				X	NA		
**Calvaruso 2018 [29]**	Italy	DAAs							X	NA	X	
**Ioannou 2018 [33]**	USA	INF/DAAs	X				X		X	NA	X	
**Pons 2020 [34]**	Spain	DAAs								X	X	
**Alonso Lopez 2020 [36]**	Spain	DAAs								X	X	
**Audureau 2020 [44]**	France	INF/DAAs	X				X	X		NA		
			**Variables included in HCC risk scores following HCV eradication or HBV control regardless of the cause of liver disease**									
			**Host factors**			**Liver disease activity**		**Cirrhosis/Fibrosis parameters**				
			**Age**	**Gender**	**Others**	**AFP**	**AST or ALT**	**PT**	**PLT**	**LSM**	**Albumin**	**Bilirubin**
**aMAP [45]**	Worldwide	All regimens	X	X					X	NA	X	X

DAAs, direct antiviral agents; IFN, interferon; LSM, liver stiffness measurement; NA: non assessed; PLT, platelets; PT, prothrombin time.

## Data Availability

Not Applicable.

## Abbreviations

AFP: alpha-fetoprotein; DAAs, direct-acting antiviral agents; HBV, hepatitis B virus; HCC, hepatocellular carcinoma; HCV, hepatitis C virus; NUCs, nucleos(t)ide analogues; HR, hazard ratio; SVR, sustained virological response; US: ultrasonography.
